# Endoscopic Ultrasound-Guided Endotherapy for Visceral Artery Pseudoaneurysm: A Case Report and Systematic Review of the Literature

**DOI:** 10.7759/cureus.111680

**Published:** 2026-06-28

**Authors:** Ajeet P Maurya, Vishal Gupta, Aman Kumar, S Sriram Kumar, Gourav Singh Rajput

**Affiliations:** 1 Department of General Surgery, All India Institute of Medical Sciences, Bhopal, IND; 2 Department of Surgical Gastroenterology, All India Institute of Medical Sciences, Bhopal, IND; 3 Department of Radiodiagnosis, All India Institute of Medical Sciences, Bhopal, IND

**Keywords:** biliary stricture, gastrointestinal bleeding, iatrogenic bile duct strictures, laparoscopic cholecystectomy complication, post-cholecystectomy bile duct injury, pseudoaneurysm of cystic artery

## Abstract

Visceral artery pseudoaneurysms (VAP) develop following inflammatory pathology or trauma. Endovascular management remains the gold standard as per the Cardiovascular and Interventional Radiological Society of Europe (CIRSE) standards. However, when endovascular therapy is not feasible, other modalities like percutaneous or endoscopic ultrasound (EUS)-guided embolization have also been utilized. Lately, EUS-guided procedures have been reported increasingly in the literature. We report a case of post-laparoscopic cholecystectomy cystic artery pseudoaneurysm complicated by associated biliary injury. It was initially managed with EUS-guided glue and coil embolization, but the patient developed recurrent bleeding with formation of a pseudoaneurysm-duodenal fistula. This complication was successfully salvaged with emergency surgery. Coexistent biliary injury was subsequently repaired with hepaticojejunostomy. We have performed a systematic review of the available literature to assess the current status of the EUS-guided endotherapy for VAP. A systematic review has been conducted following Preferred Reporting Items for Systematic Reviews and Meta-Analyses (PRISMA) and Cochrane Handbook for Systematic Reviews and Interventions guidelines.

Literature search showed 30 studies with 122 patients in whom VAP was managed by EUS-guided intervention. Studies primarily included case reports (n = 15), case series (n = 8), videos (n = 4), images (n = 2), and abstracts (n = 1). Most of the patients (108, 88.5%) were male, mean age of the cohort was 41.9 years (range: 18-73 years), chronic pancreatitis was the most common etiology, reported in 85 (69.7%) cases, splenic artery was the most commonly involved vessel, seen in 76 (61.8%) cases, and upper gastrointestinal bleeding was the most common presentation, reported in 83 (68%) cases. Unfavorable anatomy for angioembolization was the most common indication for EUS-guided endotherapy, reported in 26 (21.3%) cases, followed by failed angioembolization, which was reported in 19 (15.6%) cases. A combination of cyanoacrylate glue with coil was used in 70 (57.4%) patients during endotherapy. Pooled clinical success rate of EUS-guided endotherapy for VAP was 94.25% (95% confidence interval: 88.43-97.40%), with technical success rates approaching 100%. Pooled complication rate was 11.54% (95% CI: 6.11-19.29%), with recurrent bleeding in four (3.85%, 95% CI: 1.19-9.79%), and solid organ complications (infarct/abscess involving spleen and/or liver) in five (4.8%, 95% CI: 1.79-11.04%). Recanalization of pseudoaneurysm was noted in two patients (recurrence rate: 1.64%, 95% CI: 0.08-6.15%) on follow-up. Post-cholecystectomy cystic artery pseudoaneurysm is a rare complication. EUS-guided intervention is an evolving technique for the management of visceral artery pseudoaneurysm. There is limited and low-level evidence available at present to clearly define the role of EUS-guided endotherapy in the overall management of visceral artery pseudoaneurysm. EUS-guided intervention is a rescue therapy only for VAP at present; its first-line use (as in this case) is not supported. Endovascular management of visceral artery pseudoaneurysm remains the standard of care. Surgery remains the rescue intervention in cases where other interventions have failed.

## Introduction

Visceral artery pseudoaneurysms (VAP) are those affecting the splanchnic arteries and their branches [1]. These pseudoaneurysms are a rare entity with an incidence of 0.01-0.2% [1]. They may develop secondary to local inflammation, infection, or trauma, or may be iatrogenic [1,2]. Compared to the true aneurysm, a pseudoaneurysm develops after a contained rupture of the artery and is lined only by adventitia or by the perivascular tissues. Due to the lack of all three layers of the arterial wall, the risk of rupture is higher than that of a true aneurysm.

Because of the high risk of rupture, VAP should be managed promptly irrespective of the size or location [1]. Endovascular management remains the gold standard as per the Cardiovascular and Interventional Radiological Society of Europe (CIRSE), and surgery is occasionally deemed necessary when the endovascular intervention is not feasible, has failed, or is not available [1].

Endoscopic ultrasound (EUS) is an advanced endoscopic technique that is primarily used for diagnostic purposes. Its use, however, has been extended to certain therapeutic procedures such as pancreatic pseudocyst drainage, pancreatic necrosectomy, endoscopic biliary drainage, and celiac plexus block [3]. The use of EUS for the management of VAP has been described only recently and is evolving [4]. Unlike endovascular intervention, where the VAP is accessed through its originating artery, the approach during EUS-guided intervention is different - the VAP is accessed transluminally, usually through the stomach or duodenum.

Cystic artery pseudoaneurysm is a rare complication of laparoscopic cholecystectomy (LC); typically reported in <1% of laparoscopic cholecystectomies [5]. Thermal injury to the vessel wall by electrocautery or erosion by the metal clip is a possible cause of the formation of cystic artery pseudoaneurysm after LC [6].

Here, we present a case of a cystic artery pseudoaneurysm that developed as a complication of post-laparoscopic cholecystectomy biliovascular injury. The patient was initially managed by EUS-guided intervention but required rescue surgical intervention for a bleeding pseudoaneurysm, with subsequent surgical repair of the biliary stricture. We also conducted a systematic review of the literature to assess the current status of EUS-guided endotherapy for the management of visceral artery pseudoaneurysms.

## Case presentation

A 34-year-old male had undergone a laparoscopic cholecystectomy for symptomatic cholelithiasis at another center. Two weeks later, he developed severe upper abdominal pain radiating to the back along with melena and jaundice. He was evaluated with upper gastrointestinal endoscopy (UGIE), which was unremarkable except for duodenitis. Abdominal ultrasonography revealed a large (4.6 x 2.9 cm) cystic outpouching with arterial flow characteristics at the porta hepatis suggestive of a pseudoaneurysm. Besides, the common bile duct (CBD) was found to be dilated along with proximal intrahepatic biliary radicles. Contrast-enhanced tomography (CECT) showed a large cystic artery pseudoaneurysm (2.9 x 2.7 x 4 cm), which was managed with EUS-guided intervention. The EUS was done with a linear array echoscope; a large pseudoaneurysm was identified at the site of the cystic artery, which was punctured with a 19-G needle, and two coils (14 x 20 mm) were deployed in the pseudoaneurysm, and 2 mL of cyanoacrylate glue was also injected. An endoscopic retrograde cholangiogram (ERCP) was also performed, which showed a dilated CBD (8 mm) with multiple filling defects. A large amount of blood was removed from the CBD during ERCP, and a 10 Fr × 12 cm plastic biliary stent was placed. Post-EUS embolization CT scan showed incomplete obliteration of the pseudoaneurysm, which merely decreased in size from 2.9 x 2.7 x 4 cm to 1.6 x 1.7 x 1.4 cm, with persistent cystic artery inflow. It also showed a CBD stent in situ and a liver abscess (6.4 x 4.6 x 7 cm) in segment six.

The patient presented to us two months after the index surgery with abdominal pain and jaundice. On examination, he was hemodynamically stable but was pale and icteric. The abdomen was distended, with tenderness present over the epigastrium and umbilical area. Laboratory parameters showed severe anemia, leukocytosis, and deranged liver function tests (Table 1).

**Table 1:** Laboratory parameters of the patient at presentation to our center.

Laboratory tests	Results	Reference range	Units
Hemoglobin	8	11-15	g/dL
Total leucocyte count	13.85	4-11	10^3^/μL
Total bilirubin	13.2	0.3-1.2	mg/dL
Direct bilirubin	7.7	<0.3	mg/dL
Serum aspartate aminotransferase (AST)	76	10-50	U/L
Serum alanine aminotransferase (ALT)	40.4	10-50	U/L
Serum alkaline phosphatase (SAP)	364	40-129	U/L
Serum gamma-glutamyl transferase (GGT)	109	<60	U/L
Serum albumin	1.7	3.5-5.2	g/dL

Ultrasound (USG) showed hepatomegaly with an abscess (4.8 x 3.2 cm) in the right liver (segments six and seven). Abdominal CT angiography was performed, which showed a large (2.2 x 1.5 x 2.8 cm) persistent cystic artery pseudoaneurysm, with embolized coils within it, at the gallbladder (GB) fossa, dilated CBD with a CBD stent in situ, along with heterogeneous contents around the stent (Figures 1A-1C). In addition, multiple variable-sized liver abscesses were noted, the largest being 3.5 x 6.5 x 6.6 cm in segment six.

**Figure 1:**
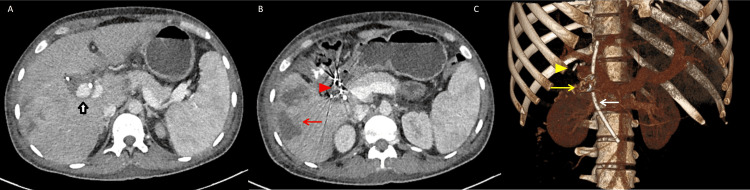
Abdominal computed tomography (CT) angiography performed before emergency surgical intervention. (A) Axial CT image showing the cystic artery pseudoaneurysm (white arrow with black outline). (B) Axial CT image showing image artifacts due to previously placed coils (red arrowhead) and liver abscess in the right lobe (red arrow). (C) 3D vascular reconstruction image showing coils in the gallbladder fossa area (yellow arrow), surgical clips previously used during cholecystectomy to secure the cystic duct and cystic artery (yellow arrowhead), and the plastic stent in the common bile duct (white arrow).

The patient was planned for angioembolization; however, he had massive hematemesis with hemodynamic instability mandating emergency surgical intervention. Intra-operatively, a large pseudoaneurysm (about 4 x 5 cm) was noted in the gallbladder fossa (Figure 2A). A cast of previously injected glue with coil was found inside the pseudoaneurysm (Figure 2B). This cast was found to have eroded the adjoining duodenum, creating a pseudoaneurysm-duodenal fistula (Figure 2C).

**Figure 2:**
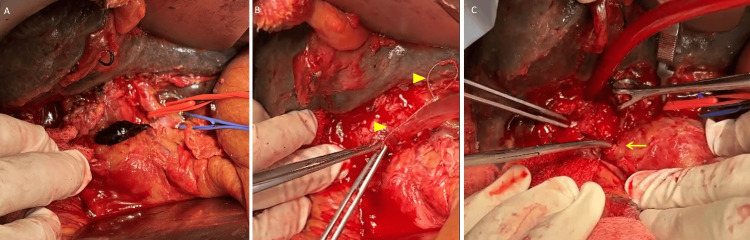
Intra-operative images during emergency abdominal exploration. (A) Pseudoaneurysm with a large clot. Proximal vascular control has been taken with a vascular sling on the proper hepatic artery (red loop) and common hepatic artery (blue loop). (B) The previously embolized coil (yellow arrowheads) is being extracted from the ruptured pseudoaneurysm. (C) Duodenal fistulous opening (yellow arrow) was noted after the cast, coil, and clots evacuation.

The lateral wall of the CBD was found to be sloughed out. After obtaining proximal vascular control, clots and glue cast with coil were evacuated, the pseudoaneurysm was ligated, the duodenal perforation site was repaired primarily, and T-tube drainage of the CBD was done as a damage control procedure (Figure 3). Feeding jejunostomy was also performed to secure nutrition post-operatively.

**Figure 3:**
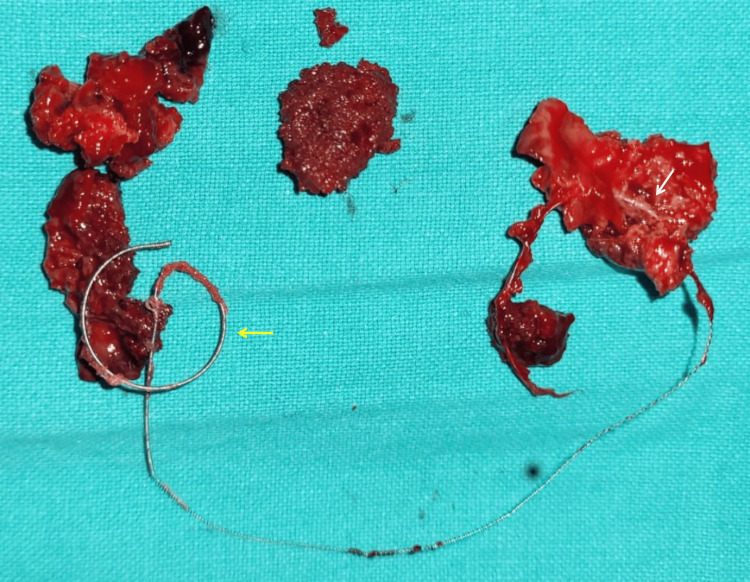
Image of pieces of glue casts with adherent clots and coil (yellow arrow), which were extracted during surgery. Coil was also seen embedded within the glue cast (white arrow).

Post-operatively, the patient had an acute upper gastrointestinal bleed after three weeks. Abdominal CT angiography revealed a new pseudoaneurysm arising from the proper hepatic artery with a size of 1.6 x 1.7 x 1.1 cm. It was managed with endovascular intervention with placement of a covered vascular stent in the hepatic artery proper covering the neck of the pseudoaneurysm resulting in its exclusion. The patient recovered subsequently with no further bleeding complications.

Subsequent evaluation revealed a Strasberg type E5 benign biliary stricture (Figures 4A, 4B). Later, biliary stricture repair was performed with Roux-en-Y hepaticojejunostomy after prior percutaneous transhepatic biliary catheterization of the excluded right posterior sectoral duct. Two separate anastomoses were used to drain the excluded right posterior sectoral duct and the strictured common hepatic duct.

**Figure 4:**
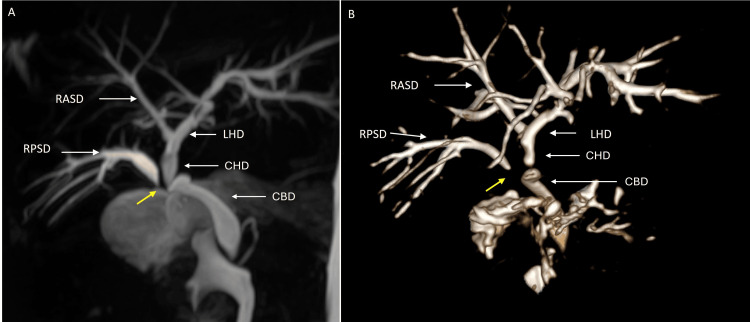
Magnetic resonance cholangiography (A) and its 3D reconstruction (B) demonstrating a Strasberg type V biliary stricture (yellow arrow). The right posterior sectoral bile duct (RPSD) is seen excluded from the rest of the extrahepatic biliary system. The common hepatic duct (CHD) was seen to be strictured below the primary confluence. The common bile duct (CBD), distal to the stricture of the common hepatic duct, was also seen. RPSD: right posterior sectoral duct; RASD: right anterior sectoral duct; LHD: left hepatic duct; CHD: common hepatic duct; CBD: common bile duct

Patient had an uneventful recovery and is doing well, being asymptomatic with normal liver function tests (total bilirubin: 0.7 mg/dL, serum alkaline phosphatase: 87 U/L, serum aspartate aminotransferase: 34 U/L, serum alanine aminotransferase: 44 U/L) at 14 months of follow-up (McDonald grade: excellent outcome).

## Discussion

Cystic artery pseudoaneurysm is a rare but grave complication of laparoscopic cholecystectomy [5]. Once identified, this vascular complication of LC requires urgent management. In a hemodynamically stable patient, angioembolization should be attempted first as per CIRSE standards, while emergency surgery is required in a hemodynamically unstable patient, as in the present case [1].

In the presence of a coexisting biliary injury, the management sequence typically follows initial management of the arterial complication (either by endovascular therapy or surgically), followed by biliary repair after several weeks. Concurrent biliary repair at the time of surgical control of an arterial injury, though it may be technically feasible, is not generally advisable due to the higher risk of post-operative complications and the risk of continued biliary ischemia and its adverse effect on the biliary repair if vascular repair is complicated by thrombotic occlusion.

In the present case, the patient had combined vascular and biliary injuries, which were managed sequentially; initial vascular control followed by surgical biliary repair. The patient had a very complicated clinical course after cholecystectomy, and finally underwent successful surgical repair of the complex biliary stricture about 10 months after recovery from all the vascular and other immediate post-cholecystectomy complications.

While the endovascular approach is the primary modality to manage cystic artery and other VAPs, primary EUS-guided intervention, directed at the cystic artery pseudoaneurysm, was not successful in the present case. Rather, it was complicated by the development of a pseudoaneurysm-duodenal fistula and recurrent bleeding. The large glue cast with coil appears to have eroded into the duodenum at the site of needle puncture. This complication likely resulted from (1) incomplete obliteration of the cystic artery source, allowing continued high-pressure inflow; (2) local tissue inflammation from the EUS procedure; and (3) the direct erosive properties of the glue material against the duodenal wall. This complex situation was successfully salvaged by emergency surgical intervention. To ascertain the current status of EUS-guided therapeutic interventions for VAP, we performed a systematic review of the literature.

Study design

The systematic review in the present study was performed as described in Preferred Reporting Items for Systematic Reviews and Meta-Analyses (PRISMA) guidelines and Cochrane Handbook for Systematic Reviews and Interventions [7].

Search strategy

A literature search was conducted using PubMed/Medline and Scopus databases up to December 2025. All the articles published in the English literature were searched using keywords “(visceral artery pseudoaneurysm, cystic artery pseudoaneurysm, hepatic artery pseudoaneurysm, gastroduodenal artery pseudoaneurysm, superior mesenteric artery pseudoaneurysm, splenic artery pseudoaneurysm, left gastric artery pseudoaneurysm) and (endoscopic ultrasound, endoscopic ultrasonography, endoscopic ultrasound guided therapy, endoscopic ultrasound guided intervention, endoscopic ultrasound guided embolization, endoscopic ultrasound guided glue injection, percutaneous ultrasound, angioembolization, embolization, EUS-guided therapy, EUS-guided intervention, EUS-guided embolization, EUS-guided glue injection)”.

Eligibility criteria

The inclusion criteria followed the PICO framework of Population, Intervention, Comparison, and Outcome. It comprised of (1) study type - all types of articles published in English language (abstract, image, video, case report, case series, and original articles); (2) study population - adult patients presenting with visceral artery pseudoaneurysm; (3) intervention - EUS-guided management of visceral artery pseudoaneurysm; and (4) outcome - success (clinical and technical) and complications of EUS-guided endotherapy for visceral artery pseudoaneurysm.

The exclusion criteria taken were (1) pediatric age group (age less than 18 years); (2) studies with visceral artery pseudoaneurysm managed by angioembolization or percutaneous glue or thrombin injection, or hybrid (EUS with percutaneous) intervention; (3) studies reporting EUS-guided vascular therapy other than for pseudoaneurysm; and (5) studies reporting only diagnostic EUS.

The primary search of literature and screening of the titles and abstracts of the studies as per eligibility criteria were performed by the first (APM) and second (VG) authors independently. Any disagreement was discussed, and a consensus was reached.

Data collection and analysis

Data collection was performed as per the PRISMA flow diagram. The extracted data included the author and year of publication, study type, number of study cohorts, age, sex, clinical presentations, primary diagnosis, site and size of VAP, indication for EUS-guided intervention, material used during EUS-guided intervention, number of attempts at EUS-guided intervention, outcomes, and post-procedural complications. Clinical success was defined as VAP-associated symptom-free discharge of the patient after intervention.

Along with the cases reported in the literature, a case of post-cholecystectomy cystic artery pseudoaneurysm managed recently at our center was also discussed. Signed informed consent was obtained from the patient before intervention and management.

Selection of studies

Primary search based on keywords identified 1491 studies. Removal of duplications, articles in non-English languages, and communications left us with 1440 studies. These records were further evaluated using abstracts, and 192 records on EUS/US-guided interventions were identified. Full-text articles were retrieved and assessed for eligibility. A total of 30 eligible studies were finally analyzed as per the inclusion criteria, as shown in the PRISMA flow diagram in Figure 5.

**Figure 5:**
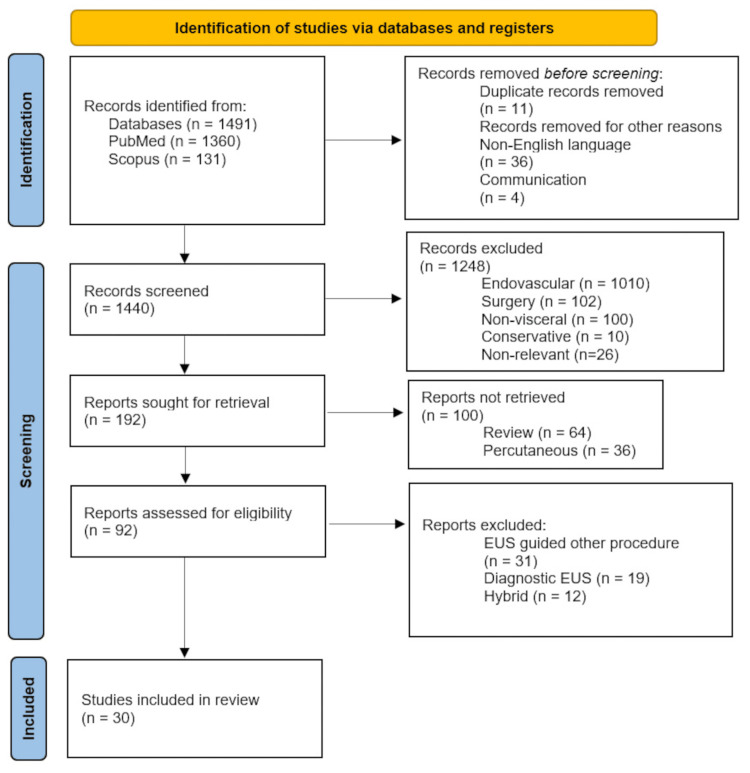
Preferred Reporting Items for Systematic Reviews and Meta-Analyses (PRISMA) flow-chart.

Characteristics of included studies

A total of 30 studies with 122 patients were finally identified fulfilling the inclusion criteria. Included studies were case reports (n = 15), case series (n = 8), videos (n = 4), images (n = 2), and abstracts (n = 1). Further details are shown in Tables 2, 3.

**Table 2:** Summary of demographic and clinical profile of patients managed with endoscopic ultrasound-guided endotherapy for visceral artery pseudoaneurysm.

Variables		n (%)
Total number of patients		122
Type of studies (n = 30)	Case reports	15 (50)
	Case series	8 (26.7)
	Videos	4 (13.3)
	Images	2 (6.7)
	Abstract	1 (3.3)
Age, years (n = 63), mean (range)		41.9 (18-73)
Gender, male		108 (88.5)
Primary disease (n = 122)	Chronic pancreatitis	85 (69.7)
	Acute pancreatitis	22 (18)
	Idiopathic	4 (3.2)
	Trauma	3 (2.5)
	Liver abscess	1 (0.8)
	Cholelithiasis	1 (0.8)
	Chronic liver disease	1 (0.8)
	Peripheral vascular disease	1 (0.8)
	Not available/specified	4 (3.2)
Presentation (n = 122)	Upper GI bleed	83 (68)
	Pain abdomen	15 (12.3)
	Asymptomatic	10 (8.2)
	Not available	14 (11.5)
Vessel involved (n = 123)	Splenic artery	76 (61.8)
	Gastroduodenal artery	21 (17.1)
	Left gastric artery	5 (4)
	Superior mesenteric artery	4 (3.2)
	Right hepatic artery	3 (2.4)
	Left hepatic artery	3 (2.4)
	Hepatic artery	2 (1.6)
	Left inferior phrenic artery	1 (0.8)
	Cystic artery	1 (0.8)
	Not specified	7 (5.7)
Size of aneurysm, cm		3.56 (0.7-9.7)
Indications (n = 122)	Unfavorable anatomy	26 (21.3)
	Failed angioembolization	19 (15.6)
	As first choice	19 (15.6)
	Hemodynamic instability	7 (5.7)
	Non-affording	6 (4.9)
	Patients’ choice	2 (1.6)
	Not mentioned	43 (35.2)
Previous treatment		15
Embolization agent (n = 122)	Thrombin	40 (32.8)
	Alcohol	2 (1.6)
	Coils	3 (2.5)
	Glue	5 (4.1)
	Coil + glue	70 (57.4)
	Thrombin + glue	1 (0.82)
	Thrombin + coil	1 (0.82)
More than one attempt (n = 122)		13 (10.65)
Outcome	Technical success	100%
	Clinical success	94.25%
Complications (n = 122)	Not mentioned	18 (14.8)
	Present (out of 104)	12 (11.54)
	Bleeding	4 (3.84)
	Pain abdomen	3 (2.88)
	Splenic infarct	2 (1.92)
	Splenic abscess	2 (1.92)
	Splenic and hepatic infarct	1 (0.96)
	No complication	92 (75.4)
Follow-up (mean, range)		22 months (1 week-101 months)

**Table 3:** Demographic and clinical characteristics of patients. CLD: chronic liver disease

S no.	Studies	Type of article	n	Age/sex	Presentation	Primary disease	Site/size of pseudoaneurysm
1	Roach et al. (2005) [4]	Case report	1	32/M	Melena	Chronic pancreatitis	Branch of superior mesenteric artery
2	Robinson et al. (2007) [8]	Case report	1	51/M	Asymptomatic	Acute pancreatitis	Splenic artery, 3 x 1.3 cm
3	Levy et al. (2008) [9]	Case series	1	67/M	Melena	Chronic pancreatitis	Superior mesenteric artery, 5 x 3 cm
4	Gonzalez et al. (2009) [10]	Case report	1	53/M	Melena	Acute on chronic pancreatitis	Splenic artery
5	Lameris et al. (2011) [11]	Case report	1	43/M	Upper GI bleed	Chronic pancreatitis	Left gastric artery, 4 x 3.2 cm
6	Chaves et al. (2012) [12]	Case report	1	29/M	Pain abdomen	Acute pancreatitis	Splenic artery, 4.5 cm
7	Roberts et al. (2012) [13]	Image	1	73/F	Pain abdomen	Peripheral vascular disease	Visceral pseudoaneurysm
8	Robb et al. (2012) [14]	Case report	1	54/M	Pain abdomen	Chronic pancreatitis	Superior mesenteric artery, 3 cm
9	Rai et al. (2014) [15]	Case report	1	25/F	Epigastric pain	Chronic pancreatitis	Splenic artery, 6.5 x 6 x 6.8 cm
10	Gamanagatti et al. (2015) [16]	Case series	3	56/M, 45/M, 30/M	Upper GI bleed	Pancreatitis (acute two, chronic one)	Gastroduodenal (n = 1), Splenic artery (n = 2)
11	Law et al. (2015) [17]	Case series	1	68/M	NA	NA	Pancreatic pseudoaneurysm, superior mesenteric artery
12	Sharma et al. (2017) [18]	Case report	1	20/M	Pain right upper abdomen	Liver abscess	Hepatic artery
13	Rai et al. (2017) [19]	Case report	1	33/M	Upper GI bleed	Chronic liver disease	Splenic artery, 3 x 2.5 cm
14	Jeffers et al. (2017) [20]	Case report	1	37/M	Melena	Chronic pancreatitis	Pancreatic neck, 8 mm
15	Gunjan et al. (2018) [21]	Case report	1	43/M	Upper GI bleed	Chronic pancreatitis	Left inferior phrenic artery, 3.6 x 2.3 cm
16	Hashimoto et al. (2018) [22]	Images	1	55/M	Upper GI bleed	Acute pancreatitis	Left gastric artery
17	Jhajharia et al. (2018) [23]	Case series	3	43/M	Pain abdomen	Chronic pancreatitis	Gastroduodenal artery, 4 x 5 cm
				25/M	Upper GI bleed	None identified	Right hepatic artery, 3 x 2.2 x 2.7 cm
				55/M	Upper GI bleed	Acute on chronic pancreatitis	Splenic artery, 1.5 x 1.3 cm
18	Rai et al. (2018) [24]	Case series	6	33/M	Upper GI bleed	Chronic pancreatitis	Splenic artery, 2 x 2.5 cm
				37/M	Upper GI bleed	Chronic pancreatitis	Splenic artery, 2 x 2.5 cm
				19/M	Upper GI bleed	Chronic pancreatitis	Splenic artery, 3 x 3 cm
				60/M	Asymptomatic	Chronic pancreatitis	Splenic artery, 4.2 x 2.7 cm
				46/M	Asymptomatic	Chronic pancreatitis	Splenic artery, 5.5 x 5.6 cm
				25/F	Asymptomatic	Chronic pancreatitis	Splenic artery, 6 x 6.5 cm
19	Sharma et al. (2019) [25]	Case report	1	50/M	Melena	Acute pancreatitis	Gastroduodenal, 4 x 6 cm
20	Sharma et al. (2019) [26]	Case report	1	52/M	Upper GI bleed	Cholelithiasis with acute cholecystitis and Mirizzi's syndrome	Cystic artery, 3.4 x 2.4 cm
21	Maharshi et al. (2020) [27]	Case series	8	35/M	-	Chronic pancreatitis	Splenic artery, 4 x 5 cm
				27/M	-	Chronic pancreatitis	Splenic artery, 4 x 4 cm
				43/M	-	Chronic pancreatitis	Splenic artery, 2.9 x 2.6 cm
				55/M	-	Chronic pancreatitis	Splenic artery, 1.8 x 1.9 cm
				30/M	-	Idiopathic	Left hepatic artery, 2 x 2 cm
				31/M	-	Idiopathic	Left hepatic artery, 2.3 x 2.2 cm
				58/M	-	Chronic pancreatitis	Gastroduodenal artery, 4 x 5 cm
				33/M	-	Chronic pancreatitis	Splenic artery, 2.9 x 2.6 cm
22	Shah et al. (2021) [28]	Images and video	1	39/M	Upper GI bleed	Acute pancreatitis	Splenic artery, 6.5 x 6.1 x 3.8 cm
23	Villa et al. (2022) [29]	Video	1	45/F	Upper GI bleed	Chronic pancreatitis with CLD	Splenic artery
24	Samanta et al. (2023) [30]	Videos	1	51/M	Upper GI bleed	Acute pancreatitis	Splenic artery, 7.2 × 6.2 × 9.7 cm
25	Hao et al. (2024) [31]	Videos	1	58/M	NA	NA	Gastroduodenal artery
26	Rai et al. (2024) [32]	Case report	1	18/M	Hemosuccus pancreaticus	Acute pancreatitis	Left gastric artery, 7.7 mm
27	Azeem et al. (2024) [33]	Case report	1	54/M	Epigastric pain	Recurrent pancreatitis	Origin not clear, near neck of pancreas, 2.9 x 2.8 cm
28	Jhajharia et al. (2024) [34]	Case series	20	57/F	Upper GI bleed	Trauma	Splenic artery, 3.2 x 3 cm
				28/M	Pain abdomen	Trauma	Right hepatic artery, 2.1 x 1.8 cm
				26/M	Pain abdomen	Chronic pancreatitis	Splenic artery, 5.5 x 3.5 cm
				40/M	Pain abdomen	Chronic pancreatitis	Splenic artery, 1.5 x 1.3 cm
				43/M	Upper GI bleed	Chronic pancreatitis	Gastroduodenal artery, 5 x 4 cm
				25/F	Upper GI bleed	Idiopathic	Right hepatic artery, 3 x 2 cm
				28/M	Pain abdomen	Chronic pancreatitis	Splenic artery, 2.8 x 2.1 cm
				36/M	Upper GI bleed	Chronic pancreatitis	Gastroduodenal artery, 1.8 x 1.2 cm
				48/M	Upper GI bleed	Chronic pancreatitis	Splenic artery, 2.2 x 1.6 cm
				34/M	Pain abdomen	Trauma	Left hepatic artery, 2.6 x 1.7 cm
				52/M	Upper GI bleed	Chronic pancreatitis	Splenic artery, 3.1 x 2.3 cm
				46/M	Upper GI bleed	Chronic pancreatitis	Splenic artery, 2.1 x 1.9 cm
				42/M	Upper GI bleed	Chronic pancreatitis	Gastroduodenal artery, 3 x 2.2 cm
				58/M	Upper GI bleed	Chronic pancreatitis	Splenic artery, 2.3 x 1.5 cm
				32/M	Pain abdomen	Chronic pancreatitis	Gastroduodenal artery, 3.9 x 1.8 cm
				39/M	Upper GI bleed	Chronic pancreatitis	Splenic artery, 2.8 x 1.9 cm
				28/M	Upper GI bleed	Chronic pancreatitis	Gastroduodenal artery, 2.6 x 2.4 cm
				45/M	Upper GI bleed	Chronic pancreatitis	Splenic artery, 5 x 3 cm
				46/M	Pain abdomen	Chronic pancreatitis	Splenic artery, 3.6 x 3 cm
				52/M	Pain abdomen	Chronic pancreatitis	Splenic artery, 4.2 x 2.6 cm
29	Samanta et al. (2024) [35]	Abstract	40	Mean: 44.4 years, male 85%	Upper GI bleed: 69.1%	Chronic pancreatitis: 75%, acute pancreatitis: 20%	Splenic artery: 67.5%, gastroduodenal artery: 22.5%. Diameter (longest: 2.78 ± 1.7 cm)
30	Rai et al. (2025) [36]	Case series	19	Mean: 38.63 years, male 89.5%	Upper GI bleed: 68.3%; asymptomatic: 31.7%	Chronic pancreatitis: 78.9%	Splenic artery: 75%, gastroduodenal artery: 10%, left gastric artery: 10%, hepatic artery: 5%. Size: mean 2.5 cm (1.5-4.1 cm)
31	Current case	Case report	1	34/M	Upper GI bleed, jaundice, abdominal pain	Post-laparoscopic cholecystectomy	Cystic artery, 2.9 x 2.7 x 4 cm

Data of individual patients were not available from two studies published as case series and abstracts with 59 patients [35,36]. One of these studies was reported as an abstract [35] and the other as a case series [36].

Demographic and clinical profile

Most patients were male (88.5%), and the mean age was 41.9 years (range: 18-73 years). The most commonly reported underlying primary disease was pancreatic in origin - chronic pancreatitis in 85 (69.67%) and acute pancreatitis in 22 (18%). Four cases (3.2%) were reported as idiopathic.

The most common presentation was upper gastrointestinal bleeding, reported in 83 (68%) cases, followed by abdominal pain, reported in 15 (12.3%) cases. A total of 10 (8.2%) patients were asymptomatic for VAP. The most affected vessel was the splenic artery, reported in 76 (61.8%) cases, followed by the gastroduodenal artery in 21 (17.1%), left gastric artery in five (4%), and superior mesenteric artery in four (3.2%) patients. The mean size of VAP was 3.56 cm (range 0.7-9.7 cm). Further details are shown in Tables 2, 3.

Indications for EUS-guided intervention

The most common indication for EUS-guided procedure was unfavorable anatomy for angioembolization as reported in 26 (21.3%) cases, followed by failed angioembolization in 19 (15.6%), hemodynamic instability in seven (5.7%), cost consideration in six (4.9%), and patients’ choice in two (1.6%) cases. EUS was used as the first choice in 19 (15.6%) cases. Indication for preferential use of EUS was not mentioned in 43 (35.2%) patients. Further details are shown in Table 4.

**Table 4:** Endoscopic ultrasound-guided endotherapy for visceral artery pseudoaneurysm: indications, interventions, and outcomes. *Bleeding unrelated to the EUS-guided intervention. DSA: digital subtraction angiography; IR: interventional radiology; PA: pseudoaneurysm; CLD: chronic liver disease

Studies	Indication	Previous treatment	Material used			Outcomes		Complication	Follow-up
			Thrombin/alcohol (dose)	Coil	Glue	Technical success	Clinical success		
Roach et al. (2005) [4]	Selective catheterization of feeding vessel not feasible	None	Thrombin (500 IU)	-	-	Yes	Yes	None	42 weeks. Rebleeding after 12 weeks. CT showed recanalization, but later re-thrombosed spontaneously.
Robinson et al. (2007) [8]	Unfavorable anatomy for angioembolization (close to celiac trifurcation)	None	Thrombin (500 IU)	-	-	Yes	Yes	None	6 weeks
Levy et al. (2008) [9]	Failed angioembolization; repeat angiography could not identify pseudoaneurysm	Angioembolization coil and gel foam	99% alcohol (7 mL)	-	-	Yes	Yes	None	16 months
Gonzalez et al. (2009) [10]	Hemodynamic instability	None	-	-	Cyanoacrylate and lipiodol (2 mL)	Yes	Yes	None	6 months
Lameris et al. (2011) [11]	Feeding vessel not identified on angiography	None	Thrombin-collagen (7 mL)	-	-	Yes	Yes	Not mentioned	10 months
Chaves et al. (2012) [12]	Not mentioned	None	Thrombin (500 IU)	-	-	Yes	Yes	Splenic infarct	4 months
Roberts et al. (2012) [13]	Failed angioembolization	Twice failed angiography	-	-	Glue and lipiodol	Yes	Yes	Not mentioned	6 weeks
Robb et al. (2012) [14]	Failed angioembolization due to unfavorable anatomy	Failed thrice radioembolization	-	Coils of various sizes	-	Yes	Yes	None reported	5 months
Rai et al. (2014) [15]	Non-affording to angioembolization	None	Thrombin (2 mL)	-	-	Yes	Yes	Not mentioned	6 months
Gamanagatti et al. (2015) [16]	One patient had previous surgical clipping; other two patients pseudoaneurysm not seen on DSA	None	Thrombin (300-500 IU)	-	-	Yes	Yes	None	1 month
Law et al. (2015) [17]	Failed angioembolization	Failed angioembolization	Alcohol (7.5 mL)	-	-	Yes	Yes	None	101 months
Sharma et al. (2017) [18]	Patient choice	None	-	First: one 10 mm coil, five 6 mm coils. Second: four 10 mm coils, three 8 mm coils	-	Yes (after two sessions)	Yes	Not mentioned	1 week
Rai et al. (2017) [19]	Not mentioned	None	-	Coil	Cyanoacrylate glue (1 mL)	Yes	Yes	Not mentioned	NA
Jeffers et al. (2018) [20]	Failed angioembolization	3 attempts? IR	-	Coils partly placed transiently but removed	-	Yes	Yes	Not mentioned	15 months
Gunjan et al. (2018) [21]	Angioembolization abandoned due to dissection of artery	Failed attempt at angioembolization and percutaneous thrombin injection	-	-	Cyanoacrylate glue (3 mL)	Yes	Yes	Not mentioned	9 months
Hashimoto et al. (2019) [22]	Invisible feeder artery	None	-	-	N‑butyl‑2‑cyanoacrylate (1 mL)	Yes	Yes	Not mentioned	2 months
Jhajharia et al. (2018) [23]	Large vessel with fear of distal embolization	None	Thrombin (1000 units)	-	-	Yes	Yes	Not mentioned	18 months
	Failed angioembolization	Failed angioembolization	Thrombin (1000 units)	-	-	Yes	Yes	Not mentioned	12 months
	Hemodynamic instability and no consent	None	Thrombin (500 IU)	-	-	Yes	Yes	Not mentioned	3 months
Rai et al. (2018) [24]	Failed angioembolization due to unfavorable anatomy	Failed radioembolization	-	Coil 8 mm	Glue (1 mL)	Yes	Yes	None	3 months
	Failed angioembolization due to unfavorable anatomy	Failed radioembolization	-	Coil 8 mm	Glue (1 mL)	Yes	Yes	None	3 months
	Failed angioembolization due to unfavorable anatomy	Failed radioembolization	-	Coil 8 mm	Glue (1 mL)	Yes	Yes	None	3 months
	Failed angioembolization due to unfavorable anatomy	Failed radioembolization	-	First: two coils (16 mm). Second: two coils (14 mm)	First: glue (1 mL). Second: glue (1 mL)	Yes (after two sessions)	Yes	None	3 months
	Failed angioembolization due to unfavorable anatomy	Failed radioembolization	-	First: three coils (16 mm). Second: two coils (16 mm)	First: glue (1 mL). Second: glue (1 mL)	Yes (after two sessions)	Yes	None	3 months
	Failed angioembolization due to unfavorable anatomy	Failed radioembolization	-	First: three coils (16 mm). Second: two coils (16 mm)	First: glue (1 mL). Second: glue (1 mL)	Yes (after two sessions)	Yes	None	3 months
Sharma et al. (2019) [25]	Patient choice	None	Second: 8 mL of human thrombin (3000 IU)	First: five 10-mm coils	-	Yes (after two sessions)	Yes	Not mentioned	2 weeks
Sharma et al. (2019) [26]	Not mentioned	None	Thrombin (500 IU)	-	-	Yes	Yes	Not mentioned	6 months
Maharshi et al. (2020) [27]	Three patients had unfavorable anatomy, while 5 refused due to cost	None	Thrombin (500 IU)	-	-	Yes	Yes	Two patients had abdominal pain.	Median: 6 months (1-9). One patient had recanalization of PA with bleed requiring repeat thrombin injection
		None	Thrombin (400 units)	-	-	Yes	Yes	-	-
		None	Thrombin (300 units)	-	-	Yes	Yes	-	-
		None	Thrombin (200 units)	-	-	Yes	Yes	-	-
		None	Thrombin (400 units)	-	-	Yes	Yes	-	-
		None	Thrombin (400 units)	-	-	Yes	Yes	-	-
		None	Thrombin (500 units)	-	-	Yes	Yes	-	-
		None	Thrombin (400 units)	-	-	Yes	Yes	-	-
Shah et al. (2021) [28]	Feeder not identified	None	-	First session: three coils. Second session: five coils	First session: glue (2 mL). Second session: glue (3 mL)	Yes (after two sessions)	Yes	Not mentioned	1 months
Villa et al. (2022) [29]	Failed angioembolization	Failed radioembolization	Thrombin (800 units)	-	Cyanoacrylate glue (0.5 mL)	Yes	Yes	Not mentioned	11 months
Samanta et al. (2023) [30]	Non-visualization on arterial phase	None	-	Four coils (14 cm x 20 mm)	Cyanoacrylate glue (4 mL)	Yes	Yes	Not mentioned	12 months
Hao et al. (2024) [31]	Failed angioembolization	Failed angioembolization	-	First: two coils. Second: two coils	First: glue (0.5 mL). Second: glue (0.5 mL)	Yes (after two sessions)	Yes	Not mentioned	NA
Rai et al. (2024) [32]	Failed angioembolization due to median arcuate ligament syndrome	Failed radioembolization	-	-	Glue (0.5 mL)	Yes	Yes	Not mentioned	3 months
Azeem et al. (2024) [33]	Origin could not be identified on angiography	Origin could not be identified	-	Two coils (14 cm x 10 mm)	2 mL of a 50/50 mixture of lipiodol and n-butyl-2-cyanoacrylate	Yes	Yes	Small splenic and hepatic embolized material	36 months
Jhajharia et al. (2024) [34]	Short neck of aneurysm	None	Thrombin (300 units)	-	-	Yes	Yes	One patient developed splenic abscess and required splenectomy	42 months
	Short neck of aneurysm	None	Thrombin (400 units)	-	-	Yes	Yes	-	50 months. One patient with CLD had upper GI bleed and died*
	Short neck of aneurysm	None	Thrombin (800 units)	-	-	Yes	Yes	-	48 months
	Short neck of aneurysm	None	Thrombin (300 units)	-	-	Yes	Yes	-	60 months
	Hemodynamic instability	None	Thrombin (800 units), two attempts	-	-	Yes (after two sessions)	Yes	-	84 months
	Short neck of aneurysm	None	Thrombin (500 units)	-	-	Yes	Yes	-	48 months
	Short neck of aneurysm	None	Thrombin (400 units)	-	-	Yes	Yes	-	52 months
	Angiographically not seen	None	Thrombin (300 units)	-	-	Yes	Yes	-	32 months
	Short neck of aneurysm	None	Thrombin (500 units)	-	-	Yes	Yes	-	4 months
	Angiographically not seen	None	Thrombin (300 units)	-	-	Yes	Yes	-	36 months
	Short neck of aneurysm	None	Thrombin (700 units)	-	-	Yes	Yes	-	62 months
	Short neck of aneurysm	None	Thrombin (400 units)	-	-	Yes	Yes	-	52 months
	Hemodynamic instability	None	Thrombin (500 units)	-	-	Yes	Yes	-	34 months
	Short neck of aneurysm	None	Thrombin (300 units)	-	-	Yes	Yes	-	46 months
	Hemodynamic instability	None	Thrombin (800 units)	-	-	Yes	Yes	-	60 months
	Short neck of aneurysm	None	Thrombin (500 units)	-	-	Yes	Yes	-	34 months
	Short neck of aneurysm	None	Thrombin (500 units)	-	-	Yes	Yes	-	28 months
	Hemodynamic instability	None	Thrombin (800 units)	-	-	Yes	Yes	-	36 months
	Short neck of aneurysm	None	Thrombin (800 units)	-	-	Yes	Yes	-	40 months
	Hemodynamic instability	None	Thrombin (500 units)	-	-	Yes	Yes	-	3 months
Samanta et al. (2024) [35]	Not mentioned	None	-	Coil	Glue (mean: 2.2 mL)	Yes, 100%. Number of sessions (mean): 1.15 ± 0.40	Yes, 90%	Splenic infarct: 1 (2.5%).	Mean: 11 months. Rebleeding on follow-up in 2 (5%), need for surgery in 1 (2.5%) patient
Rai et al. (2025) [36]	Used as first-line therapy	None	-	Coil diameter, mean: 16 mm (8-16 mm). Number of coils, median: 2 (1-4)	Glue volume median: 1 mL (1-2 mL)	Yes, 100%. Number of sessions: one: 14, two: 5, three: 1	Yes, 85% (three patients required angioembolization)	Adverse events: 2. First - splenic abscess after 4 weeks, managed by percutaneous drainage; second - abdominal pain conservatively managed	Median: 37 months (18-40 months)
Current case	Used as first-line therapy	None	-	Two coils (14 x 10 mm)	Cyanoacrylate glue (2 mL)	Yes	No (persistent GI bleed with liver abscess)	Required surgery for bleeding and hemodynamic instability	14 months

Materials used during EUS-guided intervention

Cyanoacrylate glue with coil was used in 70 (57.4%) patients. Thrombin alone was used in 40 (32.78%) patients. Cyanoacrylate glue alone was used in five (4%) patients. Thrombin with coil and thrombin with glue were used in one patient each. Coil and alcohol were used in three and two patients, respectively. Thrombin was used in a mean dose of 592.3 IU (range: 200-3500 IU). Further details are shown in Table 4.

Outcome of EUS-guided intervention

Technical and Clinical Success

Overall technical success was reported as 100% (95% CI: 96.33-100%), while pooled clinical success was 94.25% (95% CI: 88.43-97.40%). However, larger studies reported clinical success rates of 85-90%, suggesting reporting bias in smaller case reports [35,36]. Complete obliteration on the first session was achieved in 109 (89.34%) patients, while 13 (10.65%) patients required multiple sessions to achieve complete obliteration; two in 12 and three in one.

Complications

A total of 18 studies with one patient each did not mention immediate complications. In the remaining cohort of 104 patients, the overall complication rate was 11.54% (95% CI: 6.58-19.24%), with recurrent bleeding seen in four (3.85%, 95% CI: 1.19-9.79%), and solid organ complications (infarct/abscess) in five (4.8%, 95% CI: 1.79-11.04%). Splenic abscess occurred as a delayed complication in two patients (1.6% of the cohort): one required splenectomy, and the other was managed with percutaneous drainage, demonstrating that infection can occur weeks after the initial EUS intervention.

Long-term complications noted were recanalization of pseudoaneurysm in two patients (recurrent rate: 1.64%, 95% CI: 0.08-6.15%) - one underwent spontaneous thrombosis after 12 weeks and one required repeat procedure, suggesting that surveillance imaging is warranted. Further details are shown in Tables 2, 4.

Follow-Up

Mean follow-up was 22 months (range: one week - 101 months). Further details are shown in Table 4.

Subgroup Analysis

Subgroup analysis was performed to assess the effect of the material used during EUS-guided intervention on outcomes. It was found that thrombin, when used alone, was associated with both technical and clinical success rates of 100% (95% CI: 89.56-100%) and a complication rate of 12.50% (95% CI: 4.99-26.58%). No significant difference was noted between thrombin and other materials when used alone for technical success (p = 1.00), clinical success (p = 1.00), and complications (p = 0.23) (Table 5).

**Table 5:** Subgroup analysis of outcomes with respect to the type of material used during EUS-guided intervention: thrombin vs. other single agent. The data are represented as n (%). P < 0.05 was considered significant.

Outcomes	Thrombin, n = 40	Other single agent, n = 10	Test used and test value	p-Value
Technical success	40 (100)	10 (100)	Fisher's exact test	1.0
Clinical success	40 (100)	10 (100)	Fisher's exact test	1.0
Complications	5 (12.5)	0	Chi-square = 1.389	0.24

Subgroup analysis was also performed to assess the effect of the use of more than one material on outcomes. Use of any single agent was associated with both technical and clinical success rates of 100% (95% CI: 91.48-100%) and a complication rate of 10% (95% CI: 3.91-21.79%). Use of a combination of various agents was associated with a technical success rate of 100% (95% CI: 93.93-100%), a clinical success rate of 90.28% (95% CI: 80.99-95.49%), and a complication rate of 9.72% (95% CI: 4.51-19.01%). The single agent was associated with better clinical success (p = 0.023) than the combination. However, no significant difference was noted for technical success (p = 1.00) and complications (p = 0.95) (Table 6). Further subgroup analysis with respect to etiology and vessel involved could not be performed due to a lack of individual patient data in two large case series with heterogeneity within the cohort with respect to etiology and vessel involved [35,36].

**Table 6:** Subgroup analysis of outcomes with respect to single vs. combination of materials used during EUS-guided intervention. The data are represented as n (%). P < 0.05 was considered significant.

Outcomes	Single agent, n = 50	Combination, n = 72	Test used, test value	p-Value
Technical success	50 (100)	72 (100)	Fisher's exact test	1.0
Clinical success	50 (100)	65 (90.3)	Chi-square = 5.157	0.023
Complications	5 (10)	7 (9.7)	Chi-square = 0.003	0.96

Risk of bias

Risk of bias of individual studies was assessed (appendix). Case reports are unplanned studies with no stated study design. As included studies are in the form of case reports, case series, and videos/images, there might be considerable selection and publication bias. They may overestimate the success of the intervention while underestimating the associated risks and relevant outcomes. Case reports are also subject to admission and selection bias, as patients have a specific condition leading to selection for a particular procedure/treatment. There may be recall bias as well as case reports are often retrospective in nature.

Certainty of evidence was assessed using the Grading of Recommendations, Assessment, Development, and Evaluation (GRADE) framework (Table 7). Most of the included studies demonstrated good methodological reporting quality. However, all were observational studies (primarily case reports and case series), resulting in an initial low-certainty rating. The evidence was further downgraded for the risk of bias, lack of comparator groups, inconsistency in indications and complication reporting, and imprecision due to small sample sizes. Consequently, the certainty of evidence was rated as low for the technical and clinical success-related outcomes and very low for the complications-related outcomes.

**Table 7:** GRADE assessment of evidence quality. GRADE: Grading of Recommendations, Assessment, Development, and Evaluation

Outcomes	Number of participants	Findings	Certainty of evidence (GRADE)
Indications for procedure	122	Not mentioned (n = 43)	⨁◯◯◯ Very low
		Patient choice (n = 2)	
		Financial constraints/non-affordability (n = 6)	
Technical success	122	-	⨁⨁◯◯ Low
Clinical success	122	-	⨁⨁◯◯ Low
Complications	122	Outcome not reported in 18 participants/cases	⨁◯◯◯ Very low

Visceral artery pseudoaneurysms are a rare entity and most commonly involve the splenic artery (60%) and hepatic artery (20%). Involvement of the superior mesenteric (5.5%), celiac (4%), gastric and gastroepiploic (4%), jejunal, ileal, colic (3%), pancreatico-duodenal and pancreatic (2%), gastroduodenal (1.5%), and inferior mesenteric artery (<1%) is less common [37]. Cystic artery pseudoaneurysm is a rare variety of VAP [5].

Hemobilia is a rare complication of LC. It most commonly results from arterial injury, resulting in the formation of a pseudoaneurysm. In a review of 29 patients who had hemobilia after LC, pseudoaneurysm of the right hepatic artery was the most common cause of hemobilia (n = 9), while pseudoaneurysm of the cystic artery was less common (n = 3) [38]. In this review, embolization was the most common modality used to manage these VAP (23 patients). Surgery was required in five patients, and one patient required surgery after embolization.

Angioembolization is the standard of care modality to manage VAP with many advantages (less invasive, more target-specific, and less morbid) compared to surgical intervention. Percutaneous or endoscopic US-guided interventions have been reported as an alternative modality, especially when angioembolization is not feasible, e.g., deranged renal function or contrast allergy [39].

EUS-guided endotherapy for the management of gastric varices has been in practice for some time [40]. EUS-guided management of VAP, however, is a relatively new and expanded therapeutic use of EUS [4]. The current systematic literature review could find only limited studies at present (total 30 studies with 122 patients) since its first description by Roach et al., suggesting its slow adoption in clinical practice for the management of VAP [4].

As shown in this systematic review, the majority of the EUS-guided procedures have been performed for vascular complications secondary to chronic pancreatitis, which was present in 85 (69.7%) cases. The splenic artery was the most affected vessel, reported in 76 (61.8%) cases. Cystic artery pseudoaneurysm was reported in only one study [26]. In this report, cystic artery pseudoaneurysm developed secondary to acute calculous cholecystitis with Mirizzi's syndrome, and was managed with EUS-guided thrombin injection, and laparoscopic cholecystectomy was subsequently performed five days later [26]. In our case, a cystic artery pseudoaneurysm developed as a complication of cholecystectomy and was also associated with biliary injury. Upper gastrointestinal bleeding was the most common presentation reported in this systematic review, occurring in 83 (68%) cases. Our case also presented with upper gastrointestinal bleeding.

EUS-guided intervention for VAP is currently reserved as an alternate option rather than a primary modality, as shown in this systematic review; it was resorted as the first choice in only 19 (15.6%) cases. Clinical failure or technical non-feasibility of angioembolization remains the most common indication for endotherapy of VAP, as shown in this study. Unfavorable anatomy for angioembolization was the indication for EUS-guided endotherapy in 26 (21.3%) cases and failed angioembolization in 19 (15.6%) cases. In the present case, this modality was used as the first-line intervention.

A variety of materials have been used for VAP during EUS-guided endotherapy. Combination therapy is preferred over monotherapy. A combination of cyanoacrylate glue with coil has been the most common combination, and was used in 70 (57.4%) cases. A meta-analysis evaluating various agents used for EUS-guided endotherapy for gastric varices also noted that combination therapy using a coil with cyanoacrylate glue was the most commonly used material [41]. This combination has better technical and clinical success and better adverse event profile than any of the other materials used alone. However, in our analysis, we found that a single agent was superior to a combination in terms of clinical success but not in terms of technical success or complication rate. Besides, we did not find superiority of thrombin over other agents when used alone in terms of technical or clinical success and complication rate. In the present case, a combination of cyanoacrylate glue and coils was used to manage the pseudoaneurysm of the cystic artery. However, the pseudoaneurysm was only partially obliterated, and bleeding recurred.

Our review showed technical success rates of 100% and clinical success rates of 94.3% in patients undergoing EUS-guided management of VAP. All the case reports and smaller case series have reported 100% clinical success rate, while two larger studies have shown clinical success as 85% and 90% [35,36]. This may be due to reporting bias inherent in case reports.

A meta-analysis of EUS-guided management of gastric varices showed the pooled treatment efficacy rate of 93.7% (95% CI: 89.5 -96.3) [42]. Another meta-analysis of endovascular embolization for post-pancreatitis arterial pseudoaneurysm reported a technical success rate of 97% (95% CI: 92-99%) and a clinical success rate of 85% (95% CI: 81-88%) [43]. While technical success rates of EUS-guided intervention for varices and VAP may be comparable, clinical outcomes differ due to lesion hemodynamics as follows: compared to arterial pseudoaneurysms (and aneurysms), gastric varices are low-pressure vascular lesions supplied by low-flow collaterals (making embolic washout possible), whereas pseudoaneurysms are high-pressure-high-flow lesions supplied by systemic arteries (making embolic material migration and recanalization more likely without complete arterial source obliteration).

In our case, EUS-guided endotherapy was technically successful but failed clinically. Incomplete obliteration of the cystic artery pseudoaneurysm with patent inflow led to recurrent bleeding. This may be explained by the fundamental difference in the management principles. The endovascular therapy aimed to exclude the pseudoaneurysm from the arterial circulation either by occluding the pathological vessel or treating the pseudoaneurysm while preserving the flow through the pathological vessel [1]. The feeder point, i.e., the origin of the pseudoaneurysm, is occluded in either approach. The feeder point, on the other hand, appears to remain unaddressed in EUS-guided therapy, while the primary focus remains on obliteration of the main pseudoaneurysm.

Embolization of pseudoaneurysm is often complicated by inadvertent distal migration of embolic agent, leading to ischemia, infarction, and bleeding. The immediate complication rate of 6.52% has been reported after angioembolization [44]. In the present systematic review, immediate complications were not reported by 15 studies, while 20 studies reported no complications. The reported immediate procedure-related complications were minor, e.g., abdominal pain, hepatic/splenic ischemia. Long-term complications noted were recanalization of pseudoaneurysm (n = 2) with spontaneous thrombosis in one patient.

Our patient had incomplete obliteration of the pseudoaneurysm, resulting in recurrent bleeding. Pseudoaneurysm gradually refilled due to non-obliteration of its source, i.e., cystic artery. Later, the patient developed a pseudoaneurysm-duodenal fistula. This fistula most probably developed at the site of the duodenum used to inject embolic agents in the pseudoaneurysm. The cast of the glue, with the coil inside, gradually eroded into the adjoining duodenum. High intraluminal pressure within the pseudoaneurysm due to an intact arterial inflow might have also contributed to this fistulization, besides the local inflammation. Though planned, angioembolization could not be considered due to hemodynamic instability, and a salvage emergency surgery was performed with a successful outcome.

This review carries limitations as it is primarily based on case reports and case series with an inherent bias due to the absence of matched controls. There are only a limited number of cases reported since the first description of EUS-guided intervention of VAP two decades ago. Case reports are important for uncommon or unusual scenarios and often demonstrate the feasibility, safety, and effectiveness of the intervention, as shown in the present study and systematic review.

## Conclusions

Post-cholecystectomy cystic artery pseudoaneurysm is a rare complication. At present, endovascular management of VAP remains the first-line modality in hemodynamically stable patients. In patients with hemodynamic instability (as in this case), emergency surgical intervention for vascular control and subsequent definitive repair of associated biliary injury in case of combined vasculo-biliary injury is appropriate.

EUS-guided intervention is an evolving technique for the management of VAP. Although this technique is evolving, its wider adoption in routine practice is limited by the lack of wider availability of necessary expertise. However, given rapid technological advancements occurring in the field of therapeutic endoscopy, it may find its place in the armamentarium in the management of VAP. However, EUS-guided intervention is a rescue therapy only for VAP at present; its first-line use (as in this case) is not supported. There is very limited and low-level evidence available at present to clearly define and support its role in the overall management of VAP. This is primarily due to limited case numbers and low-quality studies. Further studies with large cohorts and comparative studies, including randomized controlled trials, are imperative to define the most appropriate role of EUS-guided interventions in the management of visceral artery pseudoaneurysms.

## Appendices

**Table 8:** Bias assessment of studies included in the systematic analysis.

Studies	Clear diagnosis	Clear reporting of the demography	Clear history	Adequate diagnostic tests	Confounding (other factor affecting outcome)	Indication	Intervention detail (performance bias)	Post-procedure detail	Complication reporting	Outcome reporting	Follow-up	Comment on procedure, mechanism and role
Roach et al. (2005) [4]	Yes	Yes	Yes	Yes	Yes	Yes	Yes	Yes	Yes	Yes	Yes	Yes
Robinson et al. (2007) [8]	Yes	Yes	Yes	Yes	Yes	Yes	Yes	Yes	Yes	Yes	Yes	Yes
Levy et al. (2008) [9]	Yes	Yes	Yes	Yes	Yes	Yes	Yes	Yes	Yes	Yes	Yes	Yes
Gonzalez et al. (2009) [10]	Yes	Yes	Yes	Yes	Yes	Yes	Yes	Yes	Yes	Yes	Yes	Yes
Lameris et al. (2011) [11]	Yes	Yes	Yes	Yes	Yes	Yes	Yes	Yes	No	Yes	Yes	Yes
Chaves et al. (2012) [12]	Yes	Yes	Yes	Yes	Yes	No	Yes	Yes	Yes	Yes	Yes	No
Roberts et al. (2012) [13]	Yes	Yes	Yes	Yes	Yes	Yes	Yes	No	Yes	Yes	Yes	Yes
Robb et al. (2012) [14]	Yes	Yes	Yes	Yes	Yes	Yes	Yes	No	No	Yes	Yes	Yes
Rai et al. (2014) [15]	Yes	Yes	Yes	Yes	Yes	Yes	Yes	Yes	No	Yes	Yes	No
Gamanagatti et al. (2015) [16]	Yes	Yes	Yes	Yes	Yes	Yes	Yes	Yes	Yes	Yes	Yes	Yes
Law et al. (2015) [17]	Yes	Yes	No	Yes	No	Yes	Yes	No	Yes	Yes	Yes	Yes
Sharma et al. (2017) [18]	Yes	Yes	Yes	Yes	No	Yes	Yes	No	No	Yes	No	No
Rai et al. (2017) [19]	Yes	Yes	Yes	Yes	Yes	No	Yes	No	No	Yes	Yes	No
Jeffers et al. (2018) [20]	Yes	Yes	Yes	Yes	Yes	Yes	Yes	No	No	Yes	Yes	Yes
Gunjan et al. (2018) [21]	Yes	Yes	Yes	Yes	Yes	Yes	Yes	No	No	Yes	Yes	No
Hashimoto et al. (2019) [22]	Yes	Yes	No	Yes	No	Yes	Yes	No	No	Yes	Yes	Yes
Jhajharia et al. (2018) [23]	Yes	Yes	Yes	Yes	Yes	Yes	Yes	No	No	Yes	Yes	Yes
Rai et al. (2018) [24]	Yes	Yes	Yes	Yes	Yes	Yes	Yes	Yes	Yes	Yes	Yes	Yes
Sharma et al. (2019) [25]	Yes	Yes	Yes	Yes	Yes	Yes	Yes	No	No	Yes	Yes	No
Sharma et al. (2019) [26]	Yes	Yes	Yes	Yes	Yes	No	Yes	No	Yes	Yes	Yes	Yes
Maharshi et al. (2020) [27]	Yes	Yes	Yes	Yes	Yes	Yes	Yes	Yes	Yes	Yes	Yes	Yes
Shah et al. (2021) [28]	Yes	Yes	Yes	Yes	Yes	Yes	Yes	No	No	Yes	Yes	Yes
Villa et al. (2022) [29]	Yes	Yes	Yes	Yes	Yes	Yes	Yes	No	No	Yes	Yes	No
Samanta et al. (2023) [30]	Yes	Yes	Yes	Yes	Yes	Yes	Yes	No	No	Yes	Yes	Yes
Hao et al. (2024) [31]	Yes	no	No	Yes	No	Yes	Yes	No	No	Yes	Yes	No
Rai et al. (2024) [32]	Yes	Yes	Yes	Yes	Yes	Yes	Yes	Yes	No	Yes	Yes	Yes
Azeem et al. (2024) [33]	Yes	Yes	Yes	Yes	Yes	Yes	Yes	Yes	Yes	Yes	Yes	Yes
Jhajharia et al. (2024) [34]	Yes	Yes	Yes	Yes	Yes	Yes	Yes	Yes	Yes	Yes	Yes	Yes
Samanta et al. (2024) [35]	Yes	Yes	Yes	Yes	Yes	Yes	Yes	Yes	Yes	Yes	Yes	Yes
Rai et al. (2025) [36]	Yes	Yes	Yes	Yes	Yes	Yes	Yes	Yes	Yes	Yes	Yes	Yes
